# Composition and Function of Chicken Gut Microbiota

**DOI:** 10.3390/ani10010103

**Published:** 2020-01-08

**Authors:** Ivan Rychlik

**Affiliations:** Department of Immunology, Veterinary Research Institute, 621 00 Brno, Czech Republic; rychlik@vri.cz; Tel.: +420-533-331-201

**Keywords:** chicken, gut microbiota, caecum, ileum, faecal, development, Bacteroidetes, Firmicutes

## Abstract

**Simple Summary:**

Chickens evolved for millions of years to be hatched in a nest in contact with an adult hen. However, current commercial production of chickens is based on hatching chicks in a clean hatchery environment in the absence of adult hens. The ancestors of domestic chickens inhabited a living environment different from that used for current commercial production. Currently, the lifespan of broilers is around 5 weeks, the lifespan of egg layers is around one year while chickens can live for 15–20 years. This means that studies on chicken–microbiota interactions are of specific importance. The intestinal tract of commercially hatched chicks is gradually colonised from environmental sources only, however, if the chicks are provided experimentally with microbiota from a hen they can be colonised by adult-type microbiota from the very first days of life and become resistant to infections with pathogenic *Escherichia coli*, *Clostridium perfringens*, or *Salmonella*. Because of such specificities in the interactions of chickens with their gut microbiota, current knowledge in this area is critically presented in this review.

**Abstract:**

Studies analyzing the composition of gut microbiota are quite common at present, mainly due to the rapid development of DNA sequencing technologies within the last decade. This is valid also for chickens and their gut microbiota. However, chickens represent a specific model for host–microbiota interactions since contact between parents and offspring has been completely interrupted in domesticated chickens. Nearly all studies describe microbiota of chicks from hatcheries and these chickens are considered as references and controls. In reality, such chickens represent an extreme experimental group since control chicks should be, by nature, hatched in nests in contact with the parent hen. Not properly realising this fact and utilising only 16S rRNA sequencing results means that many conclusions are of questionable biological relevance. The specifics of chicken-related gut microbiota are therefore stressed in this review together with current knowledge of the biological role of selected microbiota members. These microbiota members are then evaluated for their intended use as a form of next-generation probiotics.

## 1. Introduction

Chickens represent one of the most widespread farm animals worldwide. Domestication of chickens started approximately 5000 years ago and since that time, chicken eggs and meat have become a common source of animal protein for humans. Before domestication, chickens, as any other species, were subjected to evolution and natural selection and the domestication of chickens introduced several changes to their natural behaviour. The ancestors of domestic chickens laid eggs in nests and warmed them by heat provided by the brooding parent. Incubated eggs were in intimate contact with the adult hen for 21 days of embryonic development and the same was true for newly hatched chicks—these were in contact with the adult hen from the very first moments of their life. The ancestors of domestic chickens also inhabited and were adapted to a living environment different from that used for current commercial production. Finally, the lifespan of meat types of chickens in commercial production is around 5 weeks while chickens reach sexual maturity around week 18 of life. The lifespan of egg layers in commercial production is around one year while red jungle fowl can live for 15–20 years. These facts are commonly forgotten and ignored though correct consideration of these facts has broad consequences for chicken welfare, resistance to enteric diseases and production. Serious consideration of these facts may also prevent trivial mistakes in experimental studies on chicken microbiota. The aim of this review was therefore to remind researchers of common facts related to the physiology of chicken intestinal tract which may influence microbiota composition and to summarise current knowledge on the composition and function of the main chicken gut microbiota members.

## 2. Composition of Gut Microbiota in Adult Chickens 

**Figure 1:**
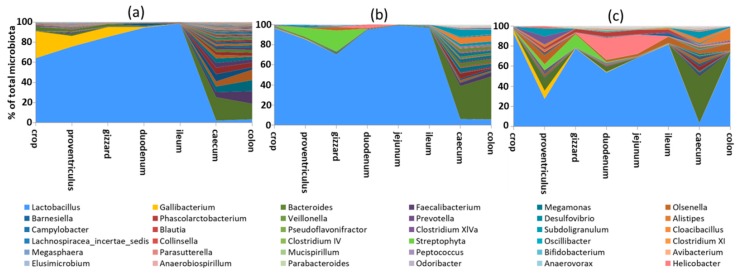
Microbiota composition along the digestive tract of 3 different adult hens. Proximal parts of the digestive tract are dominated by *Lactobacilli*. Microbiota diversity increases considerably in the caecum. Colonic microbiota can be a mixture of caecal and ileal microbiota, depending on the time of sampling related to the time of voiding the caecal content into colon. Hens (**a**,**b**) were sacrificed and sampled shortly after voiding the caecal content in the colon while hen (**c**) was sampled a longer time after voiding of the caecal content into the colon, at the time when the caecal microbiota was “washed out” and replaced with digesta originating from the ileum. Data in this figure originate from Videnska et al. [8] complemented with recent laboratory results.

Microbiota of young chickens is highly variable [1,2,3] and the microbiota of adult chickens, i.e., at least 20 weeks of age, has to be used for the definition of core chicken gut microbiota [4,5,6,7]. Proximal parts of the digestive tract of adult chickens are dominated by *Lactobacilli*, though other species are present as well [8,9]. Microbiota composition and complexity considerably increases in distal parts of the intestinal tract (caecum and colon), though colonic microbiota, due to physiology of the chicken intestinal tract, is variable and may resemble either ileal or caecal microbiota (Figure 1).

### 2.1. Crop Microbiota

Crop microbiota of adult hens is dominated by *Lactobacilli*. In addition, *Gallibacterium* (family Pasteurellaceae, phylum Proteobacteria) is characteristic of the chicken crop.

### 2.2. Stomach Microbiota

Both the proventriculus and gizzard are colonised by *Lactobacilli*, likely due to their resistance to acidic pH [8,9]. Cyanobacteria or chloroplast DNA can be found in the stomach but it is not clear whether this originates from cyanobacteria themselves or whether this DNA is of plant origin from ingested feed, the latter hypothesis being more likely than the former. Although the stomach environment is quite hostile, it is also populated by as yet uncharacterised Proteobacteria species.

### 2.3. Small Intestine Microbiota

Absolute counts of microbiota in the small intestine are rather low, around 10^5^ CFU per gram of digesta. Microbiota of the small intestine is also of quite low diversity and 50% of the total ileal microbiota can be formed by one to five genera only [10]. Microbiota composition in the duodenum, jejunum and ileum is similar to each other (Figure 1 and references [8,11]) though more thorough studies are needed to define bacterial species specifically adapted to different compartments of small intestine. Ileal microbiota can be sometimes mixed with microbiota of caecal origin [11]. Bacterial genera colonising the small intestine originate mainly from phylum Firmicutes and include *Lactobacillus*, *Enterococcus*, *Turicibacter*, *Clostridium sensu stricto* and isolates belonging to Clostridium XI cluster (family Peptostreptococcaceae, genus *Romboutsia*). *Escherichia coli* or *Helicobacter* from phylum Proteobacteria can be also found in the small intestine, the latter associated with compromised chicken performance [10].

### 2.4. Microbiota in the Caecum

Absolute counts and complexity of gut microbiota considerably increases in the caecum (Figure 1). Absolute counts of microbiota in the caecum are around 10^10^ CFU per gram of digesta and the caecum is populated by approximately 1000 different species. These belong to the two major phyla, Gram-positive Firmicutes and Gram-negative Bacteroidetes [12,13], followed by two minor phyla; Actinobacteria (Gram-positive) and Proteobacteria (Gram-negative). Firmicutes and Bacteroidetes are usually equally represented in the caecal microbiota of healthy adult hens and each form around 45% of total microbiota. The abundance of Actinobacteria and Proteobacteria is usually around 2–3% of total microbiota, though the abundance of Actinobacteria might be slightly underestimated in studies using 16S rRNA sequencing since Actinobacteria (*Olsenella*, *Collinsella* or *Bifidobacterium*) contain only between one and five copies of 16S rRNA genes [14]. Though the above-mentioned microbiota composition can be understood as a general consensus, there is high individual variation. Individuals with 10% to 90% Bacteroidetes in their microbiota exist without exhibiting any signs of abnormal behaviour. Similarly, individual chicks with more than 10% Actinobacteria and Proteobacteria can be recorded. Despite variations in abundance, representatives of these four phyla are found in the caeca of nearly all adult chickens. Besides these, there are phyla and genera which may appear in microbiota of adult hens but are not universally distributed in all individuals. These include Fusobacteria (*Fusobacterium* sp.), Elusimicrobia (*Elusimicrobium* sp.), Synergistetes (*Cloacibacillus* sp.), Spirochaetes (*Treponema* sp.) or Verrucomicrobia (*Akkermansia* sp.).

### 2.5. Colonic and Faecal Microbiota

Many studies use faecal samples for the characterisation of chicken microbiota [8,11,15,16]. Experiments, which require repeated samplings from the same bird, have to use faecal material. However, when collecting faecal samples, one should be aware of the following issues. It is not simple to force each chicken to void faecal material when needed. Investigators therefore have to collect faecal material from the floor having no control over whether the dropping was exposed to air for 10 minutes or 5 h. Since the majority of gut colonisers are strict anaerobes, this may affect final results. The composition of colonic and faecal microbiota is also considerably affected by the physiology of chicken digestion. The transition time of digesta from ingestion to excretion in chickens is as short as 2 h [17,18]. Unlike mammals such as pigs or humans, the chicken colon is quite short, only around 10 cm in adult chickens, and not much digesta is retained in the colon. After processing in the stomach, the majority of digesta passes from the small intestine to the colon and soon after is excreted in faecal droppings. This happens approximately every 2 h [19]. Only a small amount of digesta passes from ileum to the caecum where it is fermented for 8–12 h [20,21]. The caecal content is then ejected from the caecum into the colon which happens usually twice a day [22,23]. Colonic or faecal microbiota might be identical to the caecal microbiota if material is collected after caecum voiding, it can be a mixture of caecal and ileal microbiota if small intestine digesta passes through the colon immediately after voiding the caecal excretion or it can be identical to ileal microbiota if collected just prior to the new cycle of caecal contents voiding to the colon. Colonic or faecal microbiota may therefore range in composition considerably and this is a common source of variation [1,11,15,24].

### 2.6. Major Bacterial Taxa Colonising Chicken Intestinal Tract

Representatives of four major phyla colonising the chicken intestinal tract are briefly introduced in the following paragraphs.

**Figure 2:**
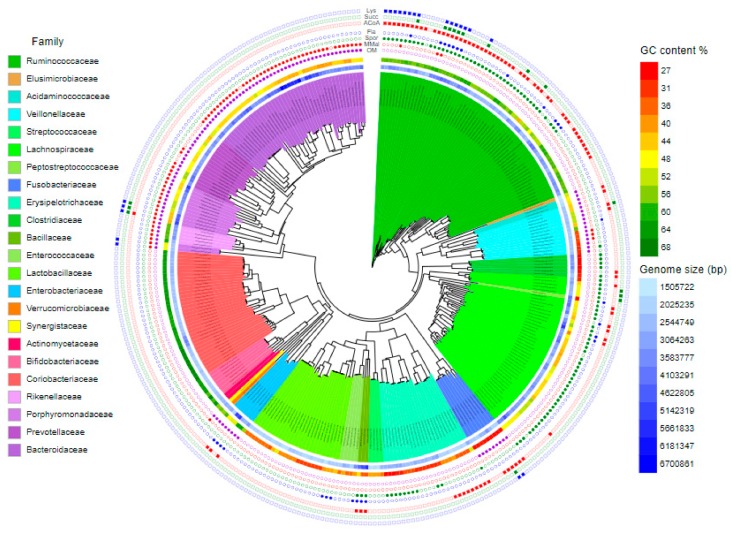
Major bacterial species colonising the chicken caecum. Genomic GC content and genome sizes are shown using a colour gradient. Families are highlighted with background colors. Presence or absence of genes for outer membrane biosynthesis (OM), succinate-methylmaloneta-propinionate pathway (MMal), spore formation (Spor), flagellar (Fla) motility and butyrate production from acetyl-CoA (ACoA), lysine (Lys) or succinate (Succ) is shown by full symbols. See Supplementary Figure S1 to zoom in. Data in this figure originate from Medvecky et al. [30] complemented with recent laboratory results.

Actinobacteria are non-spore forming, non-motile, strictly anaerobic Gram-positive bacteria characterised by high GC content (around 65%) and those colonising the intestinal tract also have a small genome size of around 2 Mbp (Figure 2). The most common colonisers belong to family Coriobacteriaceae with genera *Olsenella* and *Collinsella* and family Bifidobacteriaceae with genus *Bifidobacterium*.

Proteobacteria are non-spore forming, Gram-negative bacteria (Figure 2). Those commonly colonising the chicken caecum include both facultative (*E. coli*) and strict anaerobes (*Desulfovibrio*, *Sutterrella*, *Parasutterella*, *Anaerobiospirillum* and *Succinatomonas*). In addition, *Helicobacter* and *Campylobacter* also belong among common chicken microbiota members. Although *E. coli* is common and ubiquitous, it forms at maximum 0.1% of total caecal microbiota in healthy adults. Similarly, *Salmonella* in highly positive chickens, such as those after experimental infections, forms around 0.1% of total microbiota. This is in contrast to *Campylobacter* or *Helicobacter* which can form more than 10% of total microbiota in infected chickens [10,25]. The mode of colonisation of *E. coli* and *Salmonella* is therefore different from that used by *Helicobacter* and *Campylobacter* and measures affecting *Salmonella* colonisation may not be effective against *Campylobacter*. Unlike facultative anaerobes, strict anaerobes like *Desulfovibrio*, *Sutterrella*, *Parasutterella*, *Anaerobiospirillum* and *Succinatomonas* are genera typical of the caecal microbiota of adult hens. *Desulfovibrio* consumes free hydrogen for the reduction of sulfate thus contributing to the removal of free hydrogen formed during anaerobic fermentation in the gut environment. *Suterrella* and *Parasuterella*, similar to *Campylobacter* [26,27,28] belong to bacteria which do not utilise carbohydrates, instead, their major source of energy originates from protein, amino acid and fatty acid metabolism.

The major families from Firmicutes colonising chicken caecum include Lachnospiraceae and Ruminococcaceae, followed by Lactobacillaceae, Veillonellaceae and Erysipelotrichaceae. 

Lachnospiraceae comprise strictly anaerobic, spore forming bacteria with approximately 45% genomic GC content (Figure 2). These bacteria usually do not exhibit any specific growth and substrate preferences [29]. Although some Lachnospiraceae, e.g., *Eubacterium hallii*, *Clostridium lactatifermentans*, *Clostridium saccharolyticum*, *Clostridium clostridioforme* or *Roseburia hominis* can produce butyrate from acetyl-CoA, representatives of this family do represent the most important butyrate producers [30]. *Clostridium saccharolyticum*, *Clostridium clostridioforme* or *Roseburia hominis* may express flagella (Figure 2). *Blautia* species encode and express 5-methyltetrahydrofolate:corrinoid/iron-sulfur protein methyltransferase, acetyl-CoA synthase corrinoid activation protein and acetyl-CoA synthase corrinoid iron-sulfur protein [26], which allow them to consume CO_2_ and H_2_ to form acetate in a process called reductive acetogenesis. Similar to *Desulfovibrio*, *Blautia* is therefore important for scavenging free hydrogen released by many anaerobes during fermentation [31].

Ruminococcaceae (mainly genera *Faecalibacterium*, *Anaerotruncus*, *Butyricicoccus*, *Oscillibacter*, *Flavonifractor* or *Pseudoflavinofractor*) represent spore forming bacteria, which differ from Lachnospiraceae by a higher, around 60%, genomic GC content (Figure 2). Of these, the *Faecalibacterium* lineage lost the ability to form spores and this might be a reason why this genus developed alternative ecological adaptations and has the extra potential to consume oxygen at low concentrations [32]. Ruminococcaceae represent major butyrate producers. The majority of Ruminococcaceae produce butyrate by carbohydrate fermentation via conversion of two acetyl-CoA molecules into crotonyl-CoA [30,33,34,35]. In addition, *Flavonifractor* and *Pseudoflavinofractor* can produce butyrate also by lysine fermentation or by reduction of succinate. A lineage of *Pseudoflavinofractor* and *Anaerotruncus* represent potentially motile gut colonisers. Since vegetative cells of Ruminococcaceae and Lachnospiraceae are highly sensitive to oxygen, these bacteria are among the first ones to disappear from gut microbiota during inflammatory diseases due to the production of reactive oxygen species by macrophages and granulocytes [36,37]. In most cases, the decrease of Ruminococcaceae and Lachnospiraceae is therefore not the cause of the inflammation but its consequence [30].

Erysipelotrichaceae comprise low GC content (between 30–35% genomic GC content) and small genome (2–2.5 Mbp in size) bacteria. Spore formation is preserved among Erysipelotrichaceae but is not as widespread as in Ruminococcaceae or Lachnospiraceae (Figure 2). A lineage comprising *Faecalicoccus*, *Eubacterium cylindroides* and *Streptococcus pleomorphus* is capable of fermentation of carbohydrates to butyrate.

Lactobacillaceae are non-spore forming bacteria characterised by a low GC content and small genome. GC content of *Lactobacilli* is usually between 30–35% and genome size is around 2 Mbp in size (Figure 2). *Lactobacilli* are efficient carbohydrate fermenters, metabolism of which results in a decrease in pH and restricts the growth of other bacterial species [29]. *Lactobacillus ruminis* and *Lactobacillus agilis* encode flagellar apparatus.

Veillonellaceae and Acidaminococcaceae belong to class Negativicutes. Although these families belong among Gram-positive Firmicutes, they acquired genes for the biosynthesis of cell wall similar to that of Gram-negative bacteria. Veillonellaceae are common microbiota members with a rather small genome size, around 2.2 Mbp. However, individual genera such as *Veillonella*, *Megamonas* and *Megasphaera* have quite different biological properties. *Megamonas* is a genus with low genomic GC content of around 30%. On the other hand, the GC content in *Veillonella* and *Megasphaera* is around 44% and 53%, respectively. *Megamonas hypermegale*, but not *M. funiformis* encodes and expresses metabolic pathway genes that convert succinate to methylmalonate and propionate. This pathway is absent in *Veillonella* and *Megasphaera*. On the other hand, *Megasphaera* is a butyrate-producing bacterium utilising the most common pathway of condensation of two acetyl-CoA residues by ketoacyl thiolase into crotonyl-CoA (Figure 2).

Family Acidaminococcaceae is represented mainly by genus *Phascolarctobacterium*. *Phascolarctobacterium* belongs to bacterial species with small genomes around 1.8 Mbp in size and 48% GC content. Genes for carbohydrate metabolism are underrepresented in *Phascolarctobacterium* while genes for amino acid metabolism are abundant [26]. *Phascolarctobacterium* is capable of butyrate production. *Phascolarctobacterium* should be able also to convert succinate into methylmalonate since it encodes methylmalonyl mutase and methylmalonyl epimerase. However, as it does not encode methylmalonyl decarboxylase, the transformation of methylmalonate into propionate is unlikely.

Individual families in phylum Bacteroidetes include Rikenellaceae, Bacteroidaceae, Prevotellaceae and Porphyromonadaceae. Genomes of Bacteroidetes are bigger in size starting at 3 Mbp and commonly reaching and exceeding 6 Mbp. Representatives of all these families encode methylmalonyl epimerase, mutase and decarboxylase enabling them to produce propionate from succinate [26,38,39,40]. While this pathway is common to Rikenellaceae, Bacteroidaceae and Porphyromonadaceae, it is present only in certain lineages in Prevotellaceae.

Family Rikenellaceae and genus *Alistipes* differ from the remaining Bacteroidetes families by their high GC content between 58–60%. For yet unknown reasons *Alistipes* spp. belong among the first representatives of phylum Bacteroidetes which colonise the caecum of young chickens [4,6].

Porhyromonadaceae represent a rather heterogenous family comprising genera *Barnesiella*, *Odoribacter*, *Butyricimonas* and *Parabacteroides*. *Odoribacter* and *Butyricimonas* are capable of butyrate production via lysine fermentation and succinate reduction. *Butyricimonas* can also produce butyrate from acetyl-CoA [30].

Chicken isolates belonging to family Prevotellaceae are not characterised in detail. Isolates which we obtained in pure culture are only loosely related to *Prevotella* species characterised so far and their 16S rRNA sequences are usually only 90% similar to the closest entries in the GenBank. This suggests that chicken Prevotellaceae comprise novel genera different from those found in humans, mice or pigs. In vitro culturomics indicates that chicken Prevotellaceae are specialised in the digestion of complex polysaccharides [29], consistent with the fact that in humans Prevotellaceae are enriched in gut microbiota of humans from rural parts of Africa [41,42]. Prevotellaceae also dominate in the microbiota of adult pigs in which feed enriched for vegetable fiber is common [43,44,45].

Bacteroidaceae together with Gram-positive Lachnospiraceae and Ruminococcaceae represent the most common family characteristic of the chicken caecum. Many different species are recognised in genus *Bacteroides*. Interestingly, we recently noticed that there is a host adaptation of particular *Bacteroides* species. *B. dorei*, *B. uniformis* and *B. clarus* represented human-adapted species while *B. salanitronis*, *B. caecigallinarum* or *B. coprocola* are commonly found in chickens. For yet unknown reasons, the chicken *Bacteroides* species encode a horizontally acquired KUP gene for a predicted potassium transporter. Though in an apparent contradiction, the human-adapted species can colonise chickens, especially those of younger age [46]. Their presence in the caecum of chickens therefore indicates close contact between chickens and humans. Chicken colonisation with human-adapted species is not permanent and in adult chickens, the human-adapted *Bacteroides* species are replaced by chicken-adapted species. Genomes of Bacteroidaceae are enriched for genes involved in degradation of complex polysaccharides and their metabolism produces acetate, propionate or succinate [26,30]. Bacteroides species may acidify nutrient broths in vitro nearly as effectively as Lactobacilli. Thus, the fermentation and production of organic acid is quite extensive.

### 2.7. Other Bacteria Colonising the Chicken Intestinal Tract

Common but numerically poorly represented microbiota members belong to genera *Treponema*, *Fusobacterium*, *Akkermansia*, *Mucispirillum*, *Elusimicrobium* or *Cloacibacillus*, each of them belonging to a different phylum such as Spirochaetes, Fusobacteria, Verrucomicrobia, Defferibacteres, Elusimicrobia and Synergistetes, respectively. *Treponema* species are likely involved in fibre digestion as this is their function in termite microbiota [47] and enrichment for *Treponema* has been described also in humans from rural areas [48,49]. *Treponema* species are also more common in pigs [50,51], whose feed formula is rich in plant fibre. An abundance of Fusobacteria higher than 5% is usually an indicator of improper gut function [10].

Besides the poorly represented species, there might be lineages or particular taxa which are common but difficult to culture. Occasionally we recorded chickens who had 20% of their microbiota formed by an uncultured isolate from phylum Saccharibacteria. These bacteria are expected to be epibiotic parasites proliferating on the surface of other bacteria [52]. In addition, there are at least two new families of Clostridiales and at least one lineage of Bacteroidetes which are common to chicken microbiota but require specific, so far unknown, growth conditions [29].

## 3. How Different Is Chicken Gut Microbiota to Gut Microbiota of Other Warm-Blooded Animals? 

The basic composition of gut microbiota is similar across all omnivorous warm-blooded animals, including humans. This is due to the intestinal tract having a constant temperature between 37–42 °C, continuous nutrient supply and anaerobic environment. This is why Firmicutes and Bacteroidetes similarly dominate in human, pig and chicken gut microbiota [4,43,45,53,54,55]. In these warm-blooded species, the dominant bacterial phyla such as Firmicutes and Bacteroidetes and minority phyla such as Proteobacteria and Actinobacteria are similarly reported. There are also phyla, which may not be necessarily present in all individuals (e.g., Spirochaetes, Elusimicrobia, Synergistetes, Fusobacteria or Verrucomicrobia) and representatives of these phyla are also recorded in human, porcine and chicken gut microbiota. The composition of gut microbiota is similar down to family level. Major differences start at genus level and continue down to species level. Whenever *Faecalibacterium* is reported in chickens, its 16S rRNA sequence is only 96–97% similar to 16S rRNA of *Faecalibacterium prausnitzii* from humans. Similarly, the sequence of 16S rRNA of chicken *Megasphaera* isolates is 94–95% similar to human *Megasphaera elsdenii* and *Phascolarctobacterium* 16S rRNA sequence is only 94% similar to *Phascolarctobacterium faecium* from humans [4,24,53]. In all these examples, chicken-adapted clones likely represent novel chicken-adapted species belonging to the same genus of the already described human counterparts. Major metabolic characteristics in host-adapted species of the same genera should be the same and in agreement, both human and chicken *Megasphaera* species are efficient butyrate producers and the same is true for human and chicken *Faecalibacterium* isolates. Knowledge of the most likely function of particular microbiota members might therefore be transferred from human to porcine or chicken species. However, one has to be aware that there must be also some differences which are responsible for host adaptation. Moreover, there might be genetically minor but phenotypically significant differences between host-adapted species similar to those differentiating commensal from pathogenic *E. coli*, or antibiotic sensitive from resistant clones of otherwise identical bacterial species and clones.

## 4. Development of Chicken Gut Microbiota

### 4.1. Development of Gut Microbiota in Commercially Hatched Chickens

Development of chicken gut microbiota in commercially hatched chickens has been well described [4,16,56,57,58]. The first coloniser is represented by *E. coli* which dominates in the caecum during the first week of life. During the second week of life, *E. coli* is replaced with Gram-positive isolates from families Lachnospiraceae and Ruminococcaceae, phylum Firmicutes, with Lachnospiraceae usually appearing earlier than Ruminococcaceae. Subsequently, representatives of phylum Bacteroidetes and family Veillonellaceae (phylum Firmicutes) appear in the chicken caecum. *Alistipes* sp. from family Rikenellaceae is commonly observed as being among the first colonisers from phylum Bacteroidetes, though later during life, bacterial species from families Bacteroidaceae, Prevotellaceae and Porphyromonadaceae represent the most common Gram-negative bacteria in the chicken caecum. The principles of caecal microbiota development have been described independently by many different groups [6,16,56,57,58,59]. This also means that a comparison of gut microbiota in 4-week-old broilers and 40-week-old layers is biologically confounding and the vast majority of differences in microbiota composition have nothing to do with specifics of broilers and layers, i.e., different genetics, but mainly with different age. The described pattern of gut microbiota development is valid for chickens hatched in hatcheries and since nearly all chicks hatch in hatcheries, this mode of development may appear to be a reference. However, this mode of microbiota development is, in fact, completely artificial and has nothing in common with chicken biology. This is explained in the following paragraph.

### 4.2. Gut Microbiota Development in Chicks in Contact with Adult Hens

Chickens evolved for millions of years to be hatched in nests. If the chicks were hatched in nests, the apparent age-dependent development reported for chicks hatched in hatcheries would disappear since chicks could achieve adult-type microbiota within the first week of life. This has been described for chicks inoculated by fresh caecal or faecal extracts [24,60,61]. This has also been described for chickens fed commercial competitive exclusion products [53] and for chicks raised in the presence of adult hens [53]. The gradual development of chicken gut microbiota described for chickens from hatcheries therefore has nothing to do with age and instead, is only a function of time and likelihood of chicken exposure to a particular microbiota member. Merely because chicks are exposed to new microbiota sources with increasing age, gradual microbiota development is recorded and mistakenly associated to age. Since *E. coli* is a ubiquitously distributed facultative anaerobe, it easily survives in the environment and the likelihood that *E. coli* will come into contact with newly hatched chicks is high. The same stands for aero-tolerant *Lactobacilli* and their presence in the small intestine from the first days of life. The aerobic atmosphere of hatcheries, farms and animal houses can contain spores of Clostridiales, families Lachnospiraceae and Ruminococcaceae. This explains why Lachnospiraceae and Ruminococcaceae colonise soon after *E. coli*. The likelihood of a chicken being colonised by strict anaerobes not forming spores like those from families Veillonellaceae, Acidaminococcaceae (both Gram-positive Firmicutes), Coriobacteriaceae, Bifidobacteriaceae (both Gram-positive Actinobacteria), Rikenellaceae, Bacteroidaceae, Prevotellaceae, Porphyromonadaceae (all belonging to Gram-negative Bacteroidetes) or genera *Desulfovibrio*, *Sutterella*, *Anaerobiospirillum* or *Succinatomonas* (all belonging to Gram negative Proteobacteria) is low and therefore a longer time is needed for their appearance in gut microbiota. On the other hand, when chicks are experimentally raised in contact with an adult hen, no gradual microbiota development is recorded and chicks can adopt an adult microbiota composition within the first week of life. This does not exclude the final shaping of microbiota composition later during the chicken’s life and selection of additional microbiota members different from the parent hen microbiota but beneficial for the chick due to its diet. The meaning of this final shaping is however considerably different from the microbiota development described in commercially hatched chickens [4,62,63].

**Figure 3:**
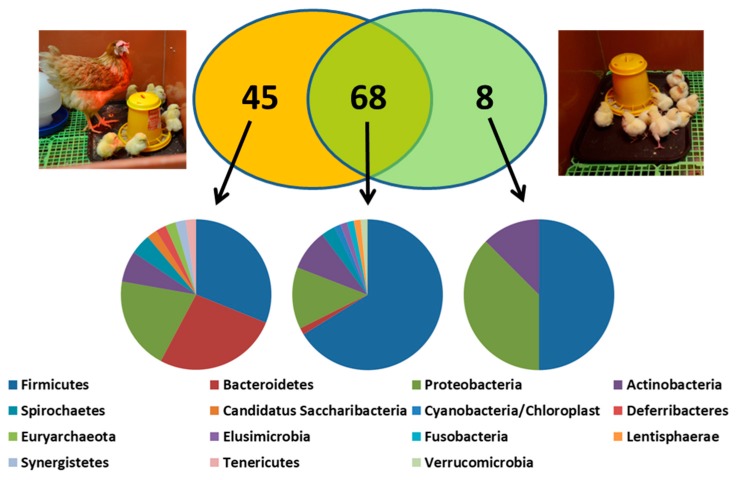
Bacterial genera of environmental or parental origin in microbiota of one-week-old chicks. Forty-five genera were more abundant in microbiota of contact chicks. These genera belonged to Bacteroidetes, Firmicutes/Veillonellaceae and Proteobacteria different from Enterobacteriaceae (left pie chart). Genera equally represented in contact and control chicks belonged mainly to Firmicutes including family Lactobacillaceae. Out of eight genera which were less abundant in contact than in control chicks, three belonged to family Enterobacteriaceae, phylum Proteobacteria. Data in this figure originate Kubasova et al. [53] complemented with recent laboratory results.

When we compared the abundance of genera in chicks raised with or without a contact hen, there were 45 genera which were more abundant in the caecal microbiota of contact chicks in comparison to control chicks (Figure 3, Table 1 and Kubasova et al. [53]). These are the genera which are underrepresented in hatcheries and chicken farms and should be provided to newly hatched chickens in the form of probiotics. Sixty-eight genera which were similarly abundant in contact and control chicks are less important for consideration as probiotics since their environmental sources are rich enough and hens do not contribute further to their transmission to chickens. Finally, three of the eight microbiota members which were more abundant in microbiota of control chickens included *E. coli*, *Proteus* and *Salmonella*. These genera are suppressed by 45 genera provided to chicks by hens. Interestingly, this is well established since the pioneering work of Nurmi and his colleagues published such findings more than 50 years ago [64].

## 5. Probiotics and Competitive Exclusion

Results obtained from the experiments with contact hens or chickens inoculated with caecal extracts clearly show which bacteria are underrepresented in microbiota of commercially hatched chickens. However, currently used probiotics are mostly based on *Lactobacilli*, *Enterococci* or *Bacilli*. *Lactobacilli* seem to be present in the environment in an amount that should fully cover the requirements of chickens [65,66,67]. *Enterococci* and *Bacilli* are less common microbiota members. Moreover, when these species are used in pure cultures for the inoculation of newly hatched chicks, they do not colonise [46]. It can be argued that even the mere passage of *Lactobacilli* may stimulate innate immunity. However, if live bacteria having a known, positive effect on gut health are used, one would expect their successful and prolonged colonisation. It should be also considered whether the positive effect of probiotics is caused directly by these bacteria or whether the positive effect can be caused by feed fermented and predigested by these bacteria. This would explain the successful use of *Lactobacilli*-fermented products like yogurts or fermented vegetables such as cabbage, and the more conflicting experience when using pure cultures of *Lactobacillus* spp. 

Unlike the sometimes-conflicting results when using probiotics consisting of a single or limited number of bacterial strains [68,69,70,71,72], there is agreement on the efficacy of commercially available competitive exclusion products such as Aviguard or Broilact in increasing chicken resistance to *Salmonella* [73,74,75,76,77]. These products contain, in addition to Lactobacillales and Clostridiales, different *Bacteroides* species, *Megamonas*, *Megasphaera*, *Dialister*, *Phascolarctobacterium*, *Sutterella*, *Parasutterella*, *Bifidobacterium* etc. [53], i.e., the same bacteria which are transferred by contact from hens to chicks. Why complex microbiota is so efficient is not understood in necessary detail. Likely factors include activation of the host immune system, competition for nutrients, production of organic acids by gut microbiota which decrease the expression of virulence factors from pathogens like Salmonella [78], the occupation of receptors and binding sites on epithelial cells and production of antimicrobial peptides. However, if *Salmonella* is inoculated in the medium together with caecal samples and this mixture is anaerobically incubated, *Salmonella* overgrows. The gut microbiota’s contribution to acidification and antimicrobial peptide production therefore appears to have little direct effect on *Salmonella*. We also observed that if nitrate is added to nutrient broths inoculated with caecal contents, *E. coli* overgrows [29]. Nitrate likely acts as an electron acceptor for anaerobic respiration of *E. coli* increasing the effectiveness of its metabolism. One of the important effects of gut microbiota therefore might be the anaerobisation of the intestinal tract environment. Bacteria, like *Faecalibacterium*, may play an important role in anaerobisation which, though highly sensitive to oxygen exposure from the atmosphere [30], may consume trace amounts of oxygen [32]. Keeping the strictly anaerobic environment free of alternative electron acceptors like nitrogen or sulfate would restrict *E. coli* or *Salmonella* from utilising the more effective anaerobic respiratory metabolism and thus prevent their overgrowth. In agreement, the induction of inflammation by *Salmonella* associated with an influx of granulocytes and macrophages to the site of infection and production of reactive oxygen species provides *Salmonella* with sulfate for anaerobic respiration [37,79].

The concept of probiotics and competitive exclusion should be revisited. New species should be tested as probiotics and the efficacy of currently available probiotics should always include the detection of the strain used for chicken inoculation in experimental but not in control chicks. Finally, use of novel bacterial species such as those belonging to genera *Megamonas*, *Megasphaera* or *Bacteroides* would also require modification of current legislation.

## 6. Common Issues in Experimental Design of Studies on Chicken Gut Microbiota

Mistakes in experimental design are rather common in studies characterising chicken gut microbiota. All of them stem from ignoring the specifics of chicken development and/or physiology. 

**Figure 4:**
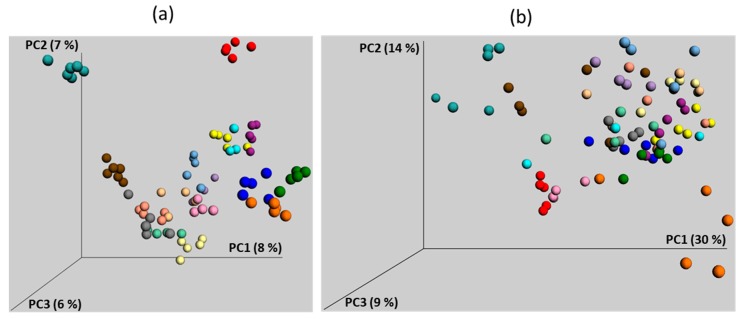
Principle Coordinate analysis (PCoA) of caecal microbiota composition in 17 groups of one-week-old control chickens. Microbiota composition was determined by sequencing of V3/V4 variable region of 16S rRNA genes as described [4]. Each dot represents one 7-day-old chick positioned in the figure based on its caecal microbiota composition. Each color represents chickens from different experiments. Both unweighted (**a**) and weighted (**b**) PCoA show experiment-dependent development of caecal microbiota. Data in this figure originate from reference [46] complemented with recent laboratory results.

First, chickens used in the vast majority of experiments are hatched in hatcheries while chickens evolved to be hatched in nests in contact with adult hens which act as a source of gut microbiota. Handling chickens from hatcheries leads to a delayed and highly variable microbiota development. In a retrospective manner, we analysed the microbiota composition in the caecum of one-week-old chickens which were used as controls in 17 completely different experiments (Figure 4). 

**Figure 5:**
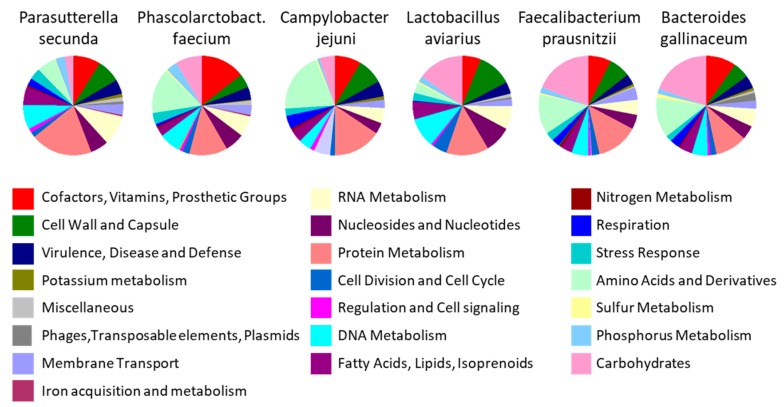
Enrichment of genomes of selected chicken gut microbiota members by genes belonging to specific functional categories. Whole genome sequences were automatically annotated by RAST (Rapid Annotation using Subsystem Technology) and predicted genes were assigned into specific functional categories. Data in this figure originate from Medvecky et al. [30] complemented with recent laboratory results.

These chicks belonged to the same genetic line, originated from the same hatchery, were housed in the same animal facilities and were given the same feed. Despite this, a clustering depending on the experiment is obvious. A similar separation was recorded when two chicken groups were housed in separated rooms [80]. If these two groups were differentially treated, random microbiota fluctuations could be mistakenly considered as the effect of tested intervention. It is therefore extremely important to critically analyse obtained results and not to rely on statistical significance only. If *Campylobacter* or *Phascolarctobacterium* increase in chickens fed with high protein diet, the biological importance of such result increases since genomes of these two species are enriched for genes for amino acid metabolism (Figure 5) [26,27,28]. However, if these species increase after diet enrichment for carbohydrates, despite statistical significance, the conclusion is likely to be biologically incorrect since these bacteria do not prefer carbohydrates. On the other hand, if *Lactobacillus* or *Faecalibacterium* increase in chickens fed a carbohydrate rich diet, this conclusion might be biologically relevant. But if these genera increase in animals on high fat or high protein diet, one should be more critical since these genera prefer carbohydrate fermentation (Figure 5 and Polansky et al. [26]). Statistical analysis of differentially abundant taxa should be therefore the first step in any experiment. This has to be followed by critical evaluation taking into consideration the known characteristics of bacteria enriched or suppressed in control and experimental groups. Repetition of experiments on chicken microbiota with chickens up to 40 days of age is strongly recommended though even this will not solve the issue completely. Should a given species be responsive to a tested intervention, e.g., feed enrichment for fat, it will be correctly identified as such if it was present in the microbiota of tested chickens prior to the experiment. But if next time this particular species is absent from the microbiota of chickens prior the repeated experiment, it cannot be detected as responsive to a high fat diet. This may happen in all animal species but is much more likely in young chickens originating from hatcheries due to their highly variable microbiota and sensitivity to colonisation [2,3,46,59].

Additional insufficiency of previously published chicken microbiota studies is administering probiotics without checking for their presence in the intestinal tract following administration [81,82,83,84,85,86]. Although it is possible that bacteria may trigger some biological processes even without colonisation, there should be a distinction between the biological effect of probiotics and their presence in the gut.

The third issue is linked with the use of litter, and faecal or caecal extracts from older chickens for the inoculation of newly hatched chicks [24,65,66]. In principle, this approach is correct. However, there are two potential issues. In some studies, faecal or caecal material from 30-day-old broilers was used. However, microbiota of commercially hatched chickens of this age is still immature and variable, and does not represent the microbiota of adult hens. Using faecal microbiota from the chickens before reaching sexual maturity may, therefore, introduce a new uncontrolled parameter— except, of course, for the studies in which the use of underdeveloped microbiota is the aim of the study. Next, when preparing caecal or faecal extracts, the extracts are commonly prepared under aerobic conditions which include resuspension, vortexing, washing and filtration that result in an efficient and repeated exposure of gut anaerobes to aerobic atmosphere with uncontrolled inactivation of different species [87,88]. Some studies included freezing aerobically prepared extracts in aliquots for future experiments [87,89]. Unfortunately, this type of storage is also aerobic. Since it is unknown how individual microbiota members survive the freezing and thawing itself, this way of handling and experimental design cannot be recommended at present. Our unpublished experience is that this type of handling promotes survival of Enterobacteriaceae and Lactobacillaceae while Bacteroidaceae, Lachnospiraceae and Ruminococcaceae are quickly lost. It can be therefore recommended to shorten the steps leading to caecal or faecal extract preparation, to use such extracts immediately and not to store them for future experiments.

An additional error is when investigators consider the age of 35 days in broilers as final, do not continue the experiments beyond this time point and draw general conclusions from chickens of this age [9,56,63,66]. Chickens reach sexual maturity around week 18 of life and may live for 15 to 20 years. Though data obtained in broilers are relevant for meat production in the poultry sector, drawing general conclusions for *Gallus gallus* when studying 35-day-old broilers is inappropriate. Similarly, using faecal microbiota of broilers as a source of gut microbiota for oral inoculation does not model the microbiota transfer from adult hens to offspring [57,66].

The last issue is associated with the specifics of digestion in chickens described in chapter 2.5. of this review. Chickens are characterised by a relatively long, bifurcated caecum and short colon (in comparison to humans or pigs). Digestion and nutrient resorption is continuous in the small intestine but resembles batch cultivation in the caecum. Faecal droppings can be of ileal or caecal origin, the former being enriched for Lactobacillaceae while the latter being enriched for Bacteroidaceae, Lachnospiraceae and Ruminococcaceae. If this is ignored and microbiota is determined in the collected faeces, or randomly collected faecal material is used for oral inoculation of young chickens, variability increases and the chickens can be clustered into different groups depending whether faecal droppings of ileal or caecal origin was collected. Although it is not possible to sacrifice chickens in all experiments, e.g., in those testing time-dependent excretion in the same animal [8], ileal and caecal digesta should be collected and analysed at the end of the experiment. Although this may appear unethical, it is ultimately better to sacrifice a small group of chickens and obtain reliable results than to obtain highly variable results with questionable meaning when collecting faecal material only.

## 7. Future Challenges

An interesting topic, which may contribute to a better definition of core chicken gut microbiota, is to analyse the microbiota of outdoor, backyard or semi-wild chickens, and compare it with microbiota of commercial hens. There are a few studies of this kind and this topic should deserve greater attention [15,90,91]. The difference in lifestyle and feed composition between indoor and outdoor chickens guarantees significant differences in microbiota composition although one must be reminded that it will not be possible to conclude that microbiota enriched in outdoor chickens will be of probiotic potential. Microbiota composition is influenced by many factors and some microbiota members from outdoor chickens may not even colonise commercial chickens on a dry, granulated diet.

There is a lack of studies on the comparison between feed, gut and litter microbiota. What is the origin of Firmicutes forming gut microbiota of chickens during the first month of life when these cannot be administered experimentally [46]? Is this feed, drinking water, human personnel or anything else? Chicken litter is not removed when raising chickens and chickens are permanently exposed to litter microbiota [65]. There are reports that litter microbiota is rich in *Lactobacilli* [65,66,67]. If this is the case, why are *Lactobacilli* based probiotics used in chicken production when the chicken’s environment is rich in *Lactobacilli*? What other bacteria from the chicken intestinal tract persist in the environment and for how long? A few studies suggested that *Faecalibacterium* can be efficiently transmitted by litter or contact [53,66,92]. These questions, which are simple to address experimentally and which have practical consequences, remain to be tested.

Though it might be personally biased, perhaps the most important objective is to generate an extensive collection of pure cultures of chicken gut anaerobes [29,30,93,94,95]. Even the finest studies on the chicken metagenome defining new species colonising the chicken intestinal tract do not allow the experiment to progress without the availability of a pure culture [31]. When pure cultures are available, whole genome sequencing of prokaryotic genomes is simple to perform and many key functions can be predicted for each isolate. Knowing the whole genomic sequence allows for identification of sequences specific to different taxa and one can use them for the design of specific PCRs. The use of strain-specific PCRs may allow tracing the fate of the tested strain in mixed and unknown populations. In addition, the isolates can be tested individually or in defined mixtures in chickens, either as potential next generation probiotics, or as starter cultures followed by testing different feed formula in pre-colonised chickens under standardised conditions. Defined mixtures can also be used in vitro to test for selective enrichment of particular taxa in differently supplemented nutrient broths. All of this is needed to complement mostly observational studies using 16S rRNA sequencing for the comparison of chicken microbiota exposed to different interventions.

## 8. Conclusions

The interactions of chickens and their microbiota is an extremely interesting area mainly due to the fact that commercial hatching completely separates chicks from contact with adult hens. This makes newly hatched chicks highly susceptible to different infections. Understanding the principles of chicken gut colonisation by bacterial microbiota may allow for the construction of a new generation of probiotics and thus improve gut health, reduce the need for antibiotic therapy and improve chicken welfare in the broadest possible terms.

## Figures and Tables

**Table 1:** List of genera which are efficiently transferred from hen to offspring. Data originated from reference [30].

Phylum	Order	Family	Transferred Genera	Non-Transferred Genera
Actinobacteria	Bifidobacteriales	Bifidobacteriaceae	*Bifidobacterium*	-
	Coriobacteriales	Coriobacteriaceae	*Olsenella, Collinsella*	*Eggerthella*
Bacteroidetes	Bacteroidales	Bacteroidaceae	*Bacteroides*	-
		Bacteroidales incertae sedis	*Phocaeicola*	-
		Porphyromonadaceae	*Parabacteroides, Barnesiella, Odoribacter, Butyricimonas, Tannerella*	
		Prevotellaceae	*Prevotella, Paraprevotella, Hallella*	-
		Rikenellaceae	*Alistipes, Rikenella*	-
Firmicutes	Clostridiales	Ruminococcaceae	*Faecalibacterium, Subdoligranulum, Gemmiger*	-
		Lachnospiraceae	*Dorea, Acetitomaculum*	*Clostridium XlVa, Ruminococcus2, Anaerostipes, Blautia*
		Peptococcaceae 1	*Peptococcus*	-
		Eubacteriaceae	*Eubacterium*	-
		Defluviitaleaceae	*Defluviitalea*	-
		Clostridiales Incertae Sedis XII	*Guggenheimella*	-
	Erysipelotrichales	Erysipelotrichaceae	*Erysipelotrichaceae incertae sedis*	-
	Selenomonadales	Acidaminococcaceae	*Phascolarctobacterium*	-
		Veillonellaceae	*Megamonas, Megasphaera, Dialister*	-
Proteobacteria	Burkholderiales	Sutterellaceae	*Sutterella, Parasutterella*	-
	Campylobacterales	Helicobacteraceae	*Helicobacter*	-
		Campylobacteraceae	*Campylobacter*	-
	Desulfovibrionales	Desulfovibrionaceae	*Desulfovibrio, Bilophila*	-
	Aeromonadales	Succinivibrionaceae	*Anaerobiospirillum, Succinatimonas, Succinivibrio*	-
	Enterobacteriales	Enterobacteriaceae	-	*Escherichia, Proteus, Salmonella*
Deferribacteres	Deferribacterales	Deferribacteraceae	*Mucispirillum*	-
Spirochaetes	Spirochaetales	Spirochaetaceae	*Treponema, Spirochaeta*	-
Synergistetes	Synergistales	Synergistaceae	*Cloacibacillus*	-
Tenericutes	Anaeroplasmatales	Anaeroplasmataceae	*Asteroleplasma*	-
Candidatus Saccharibacteria	-	-	*Saccharibacteria genera incertae sedis*	-
Euryarchaeota	Methanobacteriales	Methanobacteriaceae	*Methanobrevibacter*	-

## Supplementary Materials

The following are available online at https://www.mdpi.com/2076-2615/10/1/103/s1, Figure S1: Major bacterial species colonising the chicken caecum.
