# Supplementary figures and images for: Composition and Function of Chicken Gut Microbiota
Source: Animals (Basel). 2020 Jan 8;10(1):103. doi: 10.3390/ani10010103 (PMC7022619; doi:10.3390/ani10010103)

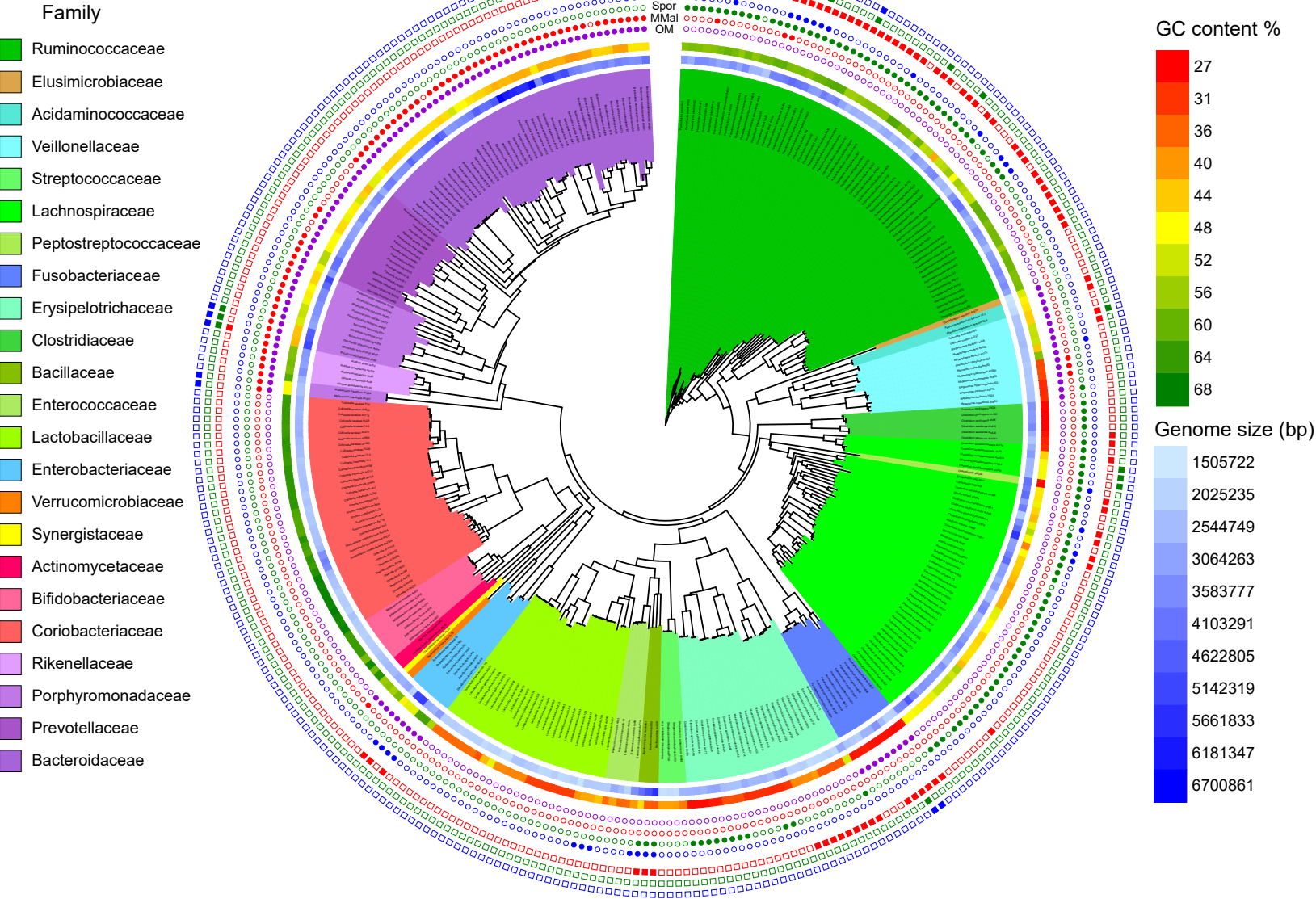

Supplement: Supplementary file 1 [file animals-10-00103-s001.zip › Figure S1.pdf]
